# Case of Fracture of the Cranium, with Protrusion of the Brain, Successfully Treated

**Published:** 1821-12

**Authors:** H. Dunn

**Affiliations:** formerly Surgeon of the Trent Hospital-Ship, and now Surgeon to the Royal Dock-yard at Woolwich.


*Case of Fracture of the Cranium, with Protrusion of the Brain, successfully treated.* By H. Dunn, M.D. formerly Surgeon of the Trent Hospital-Ship, and now Surgeon to the Royal Dock-yard at Woolwich.

FROM the singularity of the subjoined case, I think it sufficiently interesting to be submitted to the profession through the medium of the Medical and Physical Journal. It has been furnished me by my friend, Dr. Dunn, of Woolwich-yard, the author. A. C. HUTCHISON.

---

On the 31st of July, 1810, Bernard Lapatte, prisoner of war, aged about eleven years, fell from the catharpens, striking the anchor in his descent overboard, at sea, and was taken up, perfectly insensible, after a lapse of time on the water, where he remained without a struggle, and never appeared to descend below the surface. On examination, an incised wound was discovered on the scalp over the left parietal bone, completely circular, of the size of a dollar, and every portion of integument and bone so apparently detached, that, on the most minute inspection, (by report of Mr. Kelly, surgeon of the Druid,) no depressed portion of bone could be discovered. In this state the patient was admitted under my care, into the Trent hospital-ship at Cork.

The appearance of the wound was healthy, and, although comatose symptoms existed in a high degree, the disposition of the wound led us to imagine that no cause of injury, in addition to the violence of the concussion, was to be apprehended; but in a few days, every symptom became aggravated, and no time was lost in making further search for any latent injury that might exist; when, to our surprise, several fragments of bone were discovered under the level of the cranium, forced through the membrane into the substance of the brain. In this state, instant recourse was had to the operation of the trephine, as, in my opinion, collections of matter were forming under the part to be perforated, which would have a more free exit than by the fractured part at some distance; and also to afford a more ready way of removing the depressed spiculæ which occasioned this formation of matter: by which means several pieces of bone were removed, that otherwise could not have been reached, being so deeply immersed in the convolutions of the brain, that suppuration had made rapid strides in the interior; and well-formed pus was liberated in considerable quantity.

The soft parts were brought as much in contact as it was possible, and superficial dressings applied. Soon after, returning sensation gave hope of the relief which followed; but, in a few days after the operation, such was the protrusion of brain with the lacerated membrane, that no gentle means by pressure could confine them within the parietes of the cranium; and, in a few days more, a portion, the size of an egg, and resembling the half of a sand-glass, appeared strangulated. Seeing this stricture too likely to frustrate the means already undertaken to ensure success, a resolution was formed to free the exposed brain by another operation; and a longitudinal piece of the upper edge of the parietal bone was cut through, and with such immediate advantage, that a full expansion of the brain followed, and a thorough communication of heat, and consequently circulation, conveyed to the protruded portion.

During the progress of some months, the symptoms were kept manageable by the utmost attention. His youth gave every advantage to the efforts made to save him; and I have now the pleasure to report, that the brain has regained its natural position, without the slightest derangement of intellect or paralysis. His sufferings have been much heightened and prolonged by a contused wound of the thigh, which suppurated and became a fistulous ulcer: this has also been removed; and he is now enjoying such a state of health as to enable him to proceed with his father, who has uniformly conducted himself in the most correct manner, and with the most affectionate solicitude and regard for his son.

R. DUNN.

*January* 1811.

